# Philadelphia Beverage Tax and Association With Prices, Purchasing, and Individual-Level Substitution in a National Pharmacy Chain

**DOI:** 10.1001/jamanetworkopen.2023.23200

**Published:** 2023-07-13

**Authors:** Sophia V. Hua, Joshua Petimar, Nandita Mitra, Christina A. Roberto, Erica L. Kenney, Anne N. Thorndike, Eric B. Rimm, Kevin G. Volpp, Laura A. Gibson

**Affiliations:** 1 Department of Nutrition, Harvard T. H. Chan School of Public Health, Boston, Massachusetts; 2 Department of Population Medicine, Harvard Medical School and Harvard Pilgrim Health Care Institute, Boston, Massachusetts; 3 Department of Epidemiology, Harvard T. H. Chan School of Public Health, Boston, Massachusetts; 4 Department of Biostatistics, Epidemiology and Informatics, Perelman School of Medicine, University of Pennsylvania, Philadelphia; 5 Department of Medical Ethics and Health Policy, Perelman School of Medicine, University of Pennsylvania, Philadelphia; 6 Department of Medicine, Massachusetts General Hospital and Harvard Medical School, Boston

## Abstract

**Importance:**

Taxes on sweetened beverages are being implemented around the globe; an understanding of these taxes on individual-level behavior is necessary.

**Objective:**

To evaluate the degree to which the sweetened beverage tax in Philadelphia, Pennsylvania, was associated with changes in beverage prices and individual-level purchasing over time at a national pharmacy chain in Philadelphia compared with Baltimore, Maryland.

**Design, Setting, and Participants:**

Using a difference-in-differences approach and generalized linear mixed models, this cohort study examined beverage purchases made by loyalty cardholders at a national chain pharmacy retailer with stores in Philadelphia and Baltimore (control city) from before tax to after tax. Beverage sales (in US dollars) were linked by unique loyalty card numbers to enable longitudinal analyses. Data were collected from January 1, 2015, through December 31, 2017 (2 years before tax and 1 year after tax); data analyses were conducted from January through October 2022.

**Exposure:**

Implementation of Philadelphia’s 1.5 cents/oz tax on sweetened beverages.

**Main Outcomes and Measures:**

The outcomes were the change in mean beverage price per-ounce and mean beverage volume purchased per cardholder transaction. Individual-level point-of-sale scanner data from all beverage purchases were analyzed.

**Results:**

A total of 1188 unique beverages were purchased from the same stores before tax and after tax. There were 231 065 unique cardholders in Philadelphia and 82 517 in Baltimore. Mean prices of taxed beverages (n = 2 094 220) increased by 1.6 (95% CI, 1.3-2.0) cents/oz (106.7% pass-through) in Philadelphia compared with Baltimore from before tax to after tax. Philadelphia cardholders purchased 7.8% (95% CI −8.1% to −7.5%) fewer ounces of taxed beverages and 1.1% (95% CI, 0.6%-1.7%) more ounces of nontaxed beverages per transaction. Taxed beverages made up a smaller percentage of cardholders’ overall beverage purchases after tax (−13.4% [95% CI, −14.2% to −12.6%]), while nontaxed beverages made up a larger share (9.3% [95% CI, 7.7%-10.7%]).

**Conclusions and Relevance:**

In this longitudinal cohort study of the Philadelphia beverage tax, the tax was completely passed through to prices and was associated with a 7.8% decline in ounces of taxed beverages purchased at a national pharmacy chain.

## Key Points

**Question:**

Was the Philadelphia beverage tax associated with changes in beverage prices and individual-level beverage purchases among a longitudinal sample of loyalty cardholders of a large pharmacy chain in Philadelphia compared with Baltimore?

**Findings:**

In this cohort study of 313 582 loyalty cardholders, the Philadelphia beverage tax was associated with complete pass-through of the tax to prices, a 7.8% decline in the volume of taxed beverages purchased among a longitudinal cohort, and substitution of nontaxed beverages.

**Meaning:**

These findings suggest that beverage taxes are a key public health policy strategy for reducing purchases of sweetened drinks.

## Teaser

This cohort study evaluates the association of the Philadelphia beverage tax with beverage prices and purchases, including potential substitutions, using objective sales data from a large sample of customers shopping at a national pharmacy chain.

## Introduction

Sugar-sweetened beverages are the leading source of added sugars in the US diet.^1^ Consumption of these drinks is associated with excess weight gain, type 2 diabetes, and other negative health outcomes.^2,3^ For these reasons, policy makers have become interested in excise taxes on sweetened beverages to curb sugar-sweetened beverage intake while generating revenue.

Studies of sweetened beverage taxes have consistently shown that they are associated with significant increases in prices of taxed beverages and substantial declines in taxed beverage purchases.^4,5,6,7^ In Philadelphia, Pennsylvania, which implemented a 1.5 cents/oz excise tax on artificially sweetened and sugar-sweetened beverages on January 1, 2017,^8^ the mean tax pass-through (ie, the extent to which the tax is passed on to consumers through increased prices) at pharmacies has ranged from 59% to 104%.^5,9,10^ In contrast, for several cities in the San Francisco Bay Area of California, a 1 cent/oz tax led to a 45% to 77% pass-through in pharmacies.^4,11^ The declines in taxed beverage sales also vary by city from 10% in Berkeley, California, to 38% in Philadelphia.^4,5^ These results may be due to differences across the taxed cities (eg, size of the tax, sociodemographic composition) and across study designs (eg, scanner data vs surveys, different sample sizes, different periods examined).

Although a robust literature had examined tax effects using chain retailer sales data at the store level, those evaluations cannot estimate mean tax effects on individual customers or determine whether changes in purchasing are related to a changing population over time. Very few studies have examined longitudinal changes in individuals’ beverage purchases, and the existing ones are limited by small sample sizes. In 2 longitudinal studies of the Philadelphia tax using receipt collection methods, one^12^ found no statistically significant changes in adults’ purchases of taxed beverages, while the other^13^ found that the tax led to a decrease in taxed beverage purchases of 27.7%. Understanding how these taxes change individuals’ purchasing patterns is critical for understanding the degree to which these taxes may lead to health benefits, particularly in Philadelphia where, unlike in other US cities, artificially sweetened beverages (ie, generally no- or low-calorie beverages) are taxed. To address these limitations, the present study used a difference-in-differences (DID) approach to evaluate the association of the Philadelphia beverage tax with beverage prices and purchases, including potential substitutions, using objective sales data from a large sample of customers shopping at a national pharmacy chain.

## Methods

This study followed the Strengthening the Reporting of Observational Studies in Epidemiology (STROBE) reporting guideline. The University of Pennsylvania Institutional Review Board deemed this study to be non–human participants research, so there was no need for informed consent.

A national pharmacy chain provided scanner data for all packaged beverages sold from January 1, 2015, through December 31, 2017, in all stores in Philadelphia (intervention city), Baltimore, Maryland (comparison city), and Providence, Rhode Island (comparison city). Baltimore and Providence were chosen as untaxed comparison cities because they do not border Philadelphia and they have similar demographic profiles as Philadelphia.^14^ The data included beverage sales in US dollars, volume, and price information for all stock keeping units (SKUs; representing distinct items for sale). Unique card numbers linked purchases over this 3-year period either to individual loyalty cardholders or to store locations (for non–loyalty-card purchases).

Beverages were classified by tax status and beverage category. Taxed beverage categories included soda, diet soda, fruit drinks, iced tea and lemonade, sweetened sparkling water, sports drinks, sweetened coffee, and energy drinks. Nontaxed beverage categories included regular water, fruit juice, milk, sparkling water, and unsweetened coffee. Other taxed and nontaxed beverages constituted less than 1% of sales (taxed: unsweetened milk alternatives, miscellaneous taxed beverages; nontaxed: unsweetened milk alternatives, unsweetened energy drinks, unsweetened flavored waters, unsweetened iced tea or lemonades, unsweetened sports drinks, and miscellaneous nontaxed beverages). These beverages were included in main analyses of taxed and nontaxed beverages but were not separately analyzed in stratified models. Our 2 primary outcomes were the change in mean beverage price per ounce for taxed beverages, weighted by volume sold at baseline, using SKU-within-store–level data, and mean volume (by ounce) of taxed beverages purchased per transaction within individual cardholders.

Graphs of mean price per ounce and total volume sold of all taxed and nontaxed beverages in each city showed that both Baltimore and Providence had similar pretax parallel trends as Philadelphia (eFigure in Supplement 1). We relied on visual inspection instead of statistical testing for pretax parallel trends because the large size of the data set meant that even inconsequential differences in pretax trends between cities would likely be statistically significant. We compared Philadelphia with Baltimore in our primary models to allow for comparison with most of the published literature on the Philadelphia tax, which uses Baltimore as the control city. We then ran analyses comparing Philadelphia with Providence to examine whether our results were replicated with an additional control site.

Both price and volume analyses excluded the following transactions from the 16 796 831 transactions in Philadelphia and Baltimore: products that were not beverages (2819 transactions [0.02%]), beverages that we could not locate online to confirm beverage category (169 251 transactions [1.0%]), beverages whose tax status could not be determined (239 989 transactions [1.4%]), return transactions (26 372 transactions [0.2%]), beverages for which volume information was missing (18 970 transactions [0.1%]), and beverage concentrates (36 873 transactions [0.2%]). For the price-per-ounce analyses, we additionally excluded transactions for SKUs that did not appear in both the pretax and posttax periods (476 095 transactions [2.8%]) leaving 15 826 462 transactions that were aggregated into 2 963 175 week-by-store-by-SKU observations. Mean prices were weighted using pretax volume sold by each SKU in each store to estimate the effect of price changes on consumers. For the volume purchased analyses, we dropped purchases not associated with an individual loyalty card (5 104 360 [30.4%]) and purchases from loyalty card IDs that did not appear in both time periods (3 315 160 [19.7%]), leaving 7 883 037 observations that were aggregated to 6 078 488 daily transactions from 313 582 unique card holders.

### Statistical Analysis

#### Primary Analytic Models

We used generalized linear mixed models to analyze the association between tax implementation and change in mean weighted price per ounce of beverages. Our primary models included terms for store identification as a fixed effect, city, tax period, and the interaction of city by tax period (the DID estimate). Data were clustered at the SKU level and weighted by the pretax volume sold by SKU and store identification.

To analyze the association between the tax and subsequent change in mean volume of beverages purchased per card per transaction, we used cross-classified mixed models to account for imperfect nesting of loyalty cardholders and stores. Our primary models included terms for the city, tax period, the interaction of the city by tax period (DID estimate), and random intercepts for the store identification and loyalty card identification. To model relative percentage changes in volume purchased, we used generalized linear mixed models with a log link, and a negative binomial family as our outcomes were right skewed. We report percentage changes to allow for easier comparison between cities and to generate the price elasticity.

We used the Holm-Bonferroni method to adjust *P* values within families of outcomes (total taxed beverages [1 test], taxed beverage subcategories [8 tests], total nontaxed beverages [1 test], and nontaxed beverage subcategories [5 tests]). We ran 2 series of sensitivity analyses using Providence as the reference city, and including covariates for yearly quarters and percentage below the poverty line in a store’s zip code (as determined by the 2019 American Community Survey).^15^ In Providence, there were 2 440 806 week-by-store-by-SKU observations for the price-per-ounce analysis and 5 584 843 transactions from 275 335 unique card holders for the volume purchased analysis. Data were managed with Stata, version 17 (StataCorp LLC), and RStudio, version 4.2.3 (Posit PBC) was used for analysis. Two-sided *P* < .05 indicated statistical significance.

#### Secondary Analysis

We conducted 7 secondary analyses. First, we tested whether the total monthly volume of taxed beverages changed as a result of the tax (vs total transaction volume in the main analysis). We examined changes at the month level because it is possible that transaction-level reductions in the volume of taxed beverages would not necessarily mirror purchases at the month level if people simply bought the same volume of taxed beverages by making more frequent trips to the store. Second, to understand the degree to which individuals substituted nontaxed drinks, we examined changes in the percentage breakdown of taxed vs nontaxed drinks purchased per card (eg, the mean pretax transaction may have been 60% taxed beverages and 40% nontaxed beverages and then changed to 30% taxed and 70% nontaxed beverages after tax).

Third, we examined differences in taxed beverage volume purchases based on whether a cardholder made most of their purchases in stores located in low- or non–low-income zip codes. Low-income zip codes were those with at least 30% of the population at or below the federal poverty level based on the American Community Survey.^15^ Fourth, we investigated differences in the association of the tax with volume sales for high-volume purchasers (purchased in both the pretax and posttax periods and purchased in the ≥75th percentile of taxed drinks at baseline) vs low-volume purchasers (<75th percentile at baseline).

Fifth, we analyzed whether the tax differentially affected the beverage size purchased. Beverages were classified as single serving or multiserving based on the definition used by the US Food and Drug Administration for the nutrition facts panel.^16,17^ Last, to understand whether consumers were potentially going to other retailers to buy beverages, we examined whether there were changes in the number of monthly visits (sixth) and changes in the amount spent per transaction (seventh).

## Results

The chain retail pharmacy had 48 unique stores in Philadelphia and 27 in Baltimore. There were 1188 unique beverage SKUs that were purchased from the same stores before tax and after tax. In Philadelphia, 231 065 unique card holders made beverage purchases in both the pretax and posttax periods (representing 50.4% of total beverage purchases at this retailer in the city), while in Baltimore, 82 517 unique card holders made purchases in both the pretax and posttax periods (representing 42.3% of total purchases at this retailer in the city). Price analyses compared prices across the 104 weeks before tax and 52 weeks after tax. At baseline, prices in Philadelphia and Baltimore were comparable: taxed beverages had a mean (SD) price of 8.6 (6.8) and 8.2 (6.5) cents/oz, respectively, and nontaxed beverages had a mean (SD) price of 9.0 (8.1) and 9.1 (8.3) cents/oz, respectively (Table 1).

**Table 1.** DID Regression Results for Changes in Beverage Price per Ounce Following Implementation of a Beverage Tax in Philadelphia Compared With Baltimore

Beverage type^a^	Mean (SD) cents/oz^b^				Weighted DID estimate (95% CI)	*P* value^c^	Pass-through, %^d^
	Philadelphia		Baltimore				
	Before tax	After tax	Before tax	After tax			
Taxed (n = 2 094 220)^e^	8.6 (6.8)	10.3 (7.1)	8.2 (6.5)	8.6 (6.9)	1.6 (1.3 to 2.0)	<.001	106.7
Soda (n = 447 118)	5.8 (4.0)	7.3 (4.0)	5.6 (3.9)	5.8 (3.9)	1.4 (1.1 to 1.8)	<.001	93.3
Iced tea and lemonade (n = 411 840)	6.0 (3.5)	6.9 (3.4)	6.0 (3.3)	6.3 (3.4)	0.9 (0.7 to 1.1)	<.001	60.0
Fruit drinks (n = 289 643)	8.0 (5.4)	8.8 (5.1)	7.6 (5.1)	7.3 (4.6)	0.8 (0.6 to 1.0)	<.001	53.3
Sports drinks (n = 267 331)	7.0 (2.3)	9.0 (2.3)	7.1 (2.1)	7.6 (2.1)	1.8 (1.5 to 2.2)	<.001	120.0
Diet soda (n = 223 129)	5.1 (3.4)	6.6 (3.6)	5.3 (3.5)	5.6 (3.4)	1.4 (1.0 to 1.8)	<.001	93.3
Sweetened sparkling water (n = 125 332)	5.9 (5.4)	7.9 (6.5)	5.6 (4.8)	5.5 (5.1)	1.5 (1.2 to 1.8)	<.001	100.0
Sweetened coffee (n = 62 643)	20.8 (4.2)	23.2 (4.4)	20.4 (4.0)	21.3 (4.1)	1.2 (0.9 to 1.5)	<.001	80.0
Energy drinks (n = 231 614)	19.5 (5.8)	21.6 (5.3)	19.4 (6.0)	21.2 (6.6)	0.2 (−0.5 to 0.8)	.59	13.3
Nontaxed (n = 868 955)^f^	9.0 (8.1)	9.1 (7.7)	9.1 (8.3)	9.0 (7.8)	0.2 (−0.7 to 1.0)	.71	NA
Regular water (n = 318 455)	4.8 (2.8)	5.3 (3.1)	4.8 (2.8)	5.2 (3.1)	−0.002 (−0.1 to 0.1)	.97	NA
Fruit juice (n = 267 705)	14.5 (9.8)	14.3 (8.7)	14.9 (10.6)	14.7 (9.4)	0.2 (−0.3 to 0.6)	.52	NA
Milk (n = 144 939)	6.1 (4.1)	6.4 (4.1)	6.6 (4.3)	6.8 (4.8)	0.04 (−0.1 to 0.1)	.43	NA
Unsweetened sparkling water (n = 79 865)	6.2 (3.6)	6.9 (6.1)	6.3 (2.9)	6.1 (2.9)	0.5 (0.1 to 1.0)	<.05	NA
Unsweetened coffee (n = 19 815)	24.0 (8.6)	26.9 (8.3)	24.5 (9.1)	28.2 (10.4)	−1.7 (−3.5 to 0.1)	.06	NA

Abbreviations: DID, difference-in-differences; NA, not applicable.

^a^ Numbers represent the number of unique week by store identification by stock keeping unit (SKU) combinations over 104 weeks pre tax and 52 weeks post tax. In total, there were 1188 unique SKUs in this data set across 75 unique stores.

^b^ Mean prices are the mean cents-per-ounce per beverage product (SKU), per store, per week. Covariates in all regression models include store identification.

^c^ *P* < .001 indicates statistical significance after applying the Holm-Bonferroni correction for multiple testing on families of outcome (total taxed beverages [1 test], taxed beverage subcategories [8 tests], total nontaxed beverages [1 test], and nontaxed beverage subcategories [5 tests]).

^d^ The percentage pass-through was calculated for taxed beverages as the DID point estimate divided by 1.5 cents/oz.

^e^ Overall taxed category does not sum up to the composite categories because it also includes milk alternatives and other taxed beverages (n = 35 570).

^f^ Overall nontaxed category does not sum up to the composite categories because it also includes milk alternatives, flavored waters, iced tea or lemonade, sports drinks, and other nontaxed beverages (n = 35 171).

### Price Outcome

Prices of taxed beverages in Philadelphia increased by 1.6 (95% CI, 1.3-2.0) cents/oz (106.7% pass-through) compared with Baltimore after the beverage tax was implemented. This increase in price varied among taxed beverages, ranging from a 0.8 (95% CI, 0.6-1.0) cents/oz (53.3% pass-through) increase among fruit drinks to a 1.8 (95% CI, 1.5-2.2) cents/oz (120.0% pass-through) increase among sports drinks. Prices of nontaxed beverages did not differentially change in Philadelphia compared with Baltimore (Table 1). Results were similar when using Providence as the comparison city (eTable 1 in Supplement 1) and adjusting for additional covariates (eTable 2 in Supplement 1).

### Volume Outcome

Across both cities, we analyzed 3 634 736 taxed and 2 443 752 nontaxed beverage transactions for changes in volume purchased at the card level. At baseline, individual cardholders purchased fewer ounces of taxed beverages per transaction in Philadelphia relative to Baltimore (mean [SD], 73.0 [118.0] vs 90.8 [148.1] oz) and more ounces of nontaxed beverages (mean [SD], 142.3 [250.0] vs 129.4 [228.1] oz) (Table 2).

**Table 2.** DID Regression Results for Individual-Level Changes in Volume of Beverages Purchased Among Purchasers of Beverages Both Before and After Tax, Following Implementation of a Beverage Tax in Philadelphia Compared With Baltimore^a^

Beverage type^b^	Ounces purchased, mean (SD)^c^				Relative change, % (95% CI)	*P* value^d^
	Philadelphia		Baltimore			
	Before tax	After tax	Before tax	After tax		
Taxed (n = 3 634 736)^e^	73.0 (118.0)	59.1 (102.9)	90.8 (148.1)	81.5 (144.6)	−7.8 (−8.1 to −7.5)	<.001
Soda (n = 995 966)	95.5 (145.7)	75.3 (137.2)	120.4 (175.7)	103.4 (149.6)	−7.7 (−8.3 to −7.1)	<.001
Iced tea and lemonade (n = 983 266)	54.5 (79.5)	47.2 (62.6)	53.3 (78.8)	56.2 (99.6)	−12.1 (−12.7 to −11.5)	<.001
Fruit drinks (n = 551 722)	49.2 (71.4)	42.4 (57.9)	50.5 (76.4)	55.3 (123.0)	−12.6 (−13.3 to −11.9)	<.001
Sports drinks (n = 448 602)	43.1 (35.4)	39.4 (26.8)	43.8 (34.6)	42.5 (31.1)	−5.0 (−5.8 to −4.2)	<.001
Diet soda (n = 487 937)	96.3 (122.8)	75.9 (94.7)	122.5 (157.4)	102.1 (134.0)	−5.0 (−5.9 to −4.2)	<.001
Sweetened sparkling water (n = 283 874)	54.2 (65.1)	51.6 (64.5)	45.6 (54.7)	46.5 (55.3)	−3.8 (−5.1 to −2.4)	<.001
Sweetened coffee (n = 100 391)	18.5 (12.4)	17.0 (10.0)	19.7 (15.9)	19.7 (13.9)	−6.8 (−8.1 to −5.5)	<.001
Energy drinks (n = 345 044)	21.4 (14.9)	20.8 (14.3)	21.0 (14.4)	21.0 (12.7)	−2.5 (−3.3 to −1.7)	<.001
Nontaxed (n = 2 443 752)^f^	142.3 (250.0)	134.0 (243.0)	129.4 (228.1)	118.7 (222.8)	1.1 (0.6 to 1.7)	<.001
Regular water (n = 1 108 582)	227.0 (342.1)	206.3 (334.0)	203.4 (316.5)	177.6 (307.4)	2.2 (1.4 to 3.1)	<.001
Fruit juice (n = 426 901)	37.4 (34.0)	39.1 (32.6)	38.6 (51.6)	39.3 (49.1)	1.9 (0.8 to 2.9)	<.001
Milk (n = 773 300)	86.9 (57.7)	85.4 (56.8)	90.8 (88.7)	83.2 (74.9)	2.5 (2.0 to 3.1)	<.001
Unsweetened sparkling water (n = 210 764)	44.5 (39.9)	50.3 (42.0)	39.2 (36.9)	49.2 (44.3)	−4.1 (−5.8 to −2.4)	<.001
Unsweetened coffee (n = 46 915)	12.9 (8.4)	12.7 (8.4)	12.7 (7.1)	12.8 (7.6)	−2.5 (−5.0 to −0.004)	<.05

Abbreviation: DID, difference-in-differences.

^a^ Generalized linear mixed models with a log link were used to produce results for relative percentage changes in volume. Baltimore was the comparison city.

^b^ Numbers represent the number of unique transactions over 104 weeks pre tax and 52 weeks after tax.

^c^ Mean ounces purchased are raw means per card, per transaction. Regression models only include cardholder IDs that had beverage transactions in both the pretax and posttax period. To convert ounces to grams, multiply by 28.

^d^ *P* < .05 indicates statistical significance after applying the Holm-Bonferroni correction for multiple testing on families of outcomes (total taxed beverages [1 test], taxed beverage subcategories [8 tests], total nontaxed beverages [1 test], and nontaxed beverage subcategories [5 tests]).

^e^ The number of observations in the overall taxed category are fewer than the sum of the composite categories because it sums the volume of all taxed beverages purchased in an individual transaction, creating a single observation if there were multiple taxed beverage types included in the overall analysis.

^f^ The number of observations in the overall nontaxed category are fewer than the sum of the composite categories because it sums the volume of all nontaxed beverages purchased in an individual transaction, creating a single observation if there were multiple nontaxed beverage types included in the overall analysis.

The tax was associated with a 7.8% (95% CI, −8.1% to −7.5%) decline in ounces of taxed beverages purchased per transaction among cardholders in Philadelphia vs Baltimore. The decreases in purchases by beverage category ranged from −2.5% (95% CI, −3.3% to −1.7%) for energy drinks to −12.6% (95% CI, −13.3% to −11.9%) for fruit drinks (Table 2).

Cardholders in Philadelphia also increased their purchasing of nontaxed beverage ounces by 1.1% (95% CI, 0.6%-1.7%) compared with Baltimore after tax. Cardholders increased purchases of regular water by 2.2% (95% CI, 1.4%-3.1%), milk by 2.5% (95% CI, 2.0%-3.1%), and fruit juice by 1.9% (95% CI, 0.8%-2.9%), but decreased purchases of nontaxed sparkling water by −4.1% (95% CI, −5.8% to −2.4%) and nontaxed coffee by −2.5% (95% CI, −5.0% to −0.004%) (Table 2). Results were similar using Providence as the comparison city (eTable 3 in Supplement 1) and adjusting for additional covariates (eTable 4 in Supplement 1) with the exception of some variation for certain nontaxed beverage categories.

### Secondary Analysis

The total volume of taxed beverages purchased per card per month decreased by 8.2% (95% CI, −8.7% to −7.8%) and the volume of nontaxed beverages purchased per card per month increased by 1.0% (95% CI, 0.3%-1.7%) in Philadelphia compared with Baltimore after tax (eTable 5 in Supplement 1). The percentage of cardholders’ total beverage purchases in ounces that were taxed decreased by 13.4% (95% CI, −14.2% to −12.6%) and the percentage that were nontaxed increased by 9.3% (95% CI, 7.7%-10.7%) in Philadelphia compared with Baltimore after tax (eTable 6 in Supplement 1).

Although beverage prices did not differ based on a store’s zip code, the mean volume of taxed beverages purchased per card per transaction in Philadelphia compared with Baltimore decreased by −12.7% (95% CI, −14.3% to −10.9%) after tax for participants who shopped primarily in stores located in low-income zip codes. This was significantly more than the −7.3% (95% CI, −7.6% to −7.0%) decrease for participants who shopped primarily in stores located in non–low-income zip codes. There were no zip code differences in volume purchased of nontaxed beverages (eTable 7 in Supplement 1).

The mean volume of taxed and nontaxed beverages purchased per card per transaction was not significantly modified by the amount of taxed beverages individuals purchased at baseline (eTable 8 in Supplement 1). The percentage of participants’ purchases of taxed beverages in multiserving containers decreased (−18.4% [95% CI, −19.3% to −17.4%]), while the percentage for nontaxed beverages increased (15.4% [95% CI, 13.7%-17.0%]) when comparing Philadelphia with Baltimore after tax (eTable 9 in Supplement 1). Finally, the number of monthly visits per person before tax to after tax in Philadelphia compared with Baltimore did not change (−0.001 [95% CI, −0.03 to 0.03]) and consumers spent more per transaction (7.0 [95% CI, 6.7-9.7] cents).

## Discussion

This longitudinal cohort study used data from a national pharmacy chain that indicated that the 1.5 cents/oz sweetened beverage tax in Philadelphia led to 106.7% pass-through of the tax to consumers, which was associated with a 7.8% reduction in ounces of taxed beverages purchased per transaction. These effects were larger for customers who shopped primarily in stores located in low-income zip codes vs stores in non–low-income zip codes, even though the price per ounce of beverages was similar across zip codes. The tax was also associated with substitution away from taxed beverages to nontaxed water, fruit juice, and milk.

Our tax pass-through results are similar to those of other studies that estimated pass-through at pharmacies in Philadelphia using chain retailer data (95%-104%),^5,10^ but differ from a study estimating pass-through to be 59% among a smaller number of Philadelphia pharmacies.^9^ The observed pass-through in our study is also higher than estimated pass-through in pharmacies located in the San Francisco Bay Area (45%-77%).^4,11^ These differing results may be driven by differences in tax size, store types studied, and research design used. These pass-through estimates are greater than the observed pass-through at Philadelphia supermarkets (43%) and mass merchandisers (58%), but less than convenience stores with gas stations (185%) and independent corner stores (120%).^5,6,9^

Our estimates for reductions in taxed beverage sale volume at the individual level are slightly lower than estimated declines using chain retailer sales data at the store level for multiple pharmacies in Philadelphia (13%).^5^ Our results also revealed that Philadelphia shoppers (compared with those in Baltimore) substituted taxed beverages with nontaxed water, 100% juice, and milk after the tax. Because switching to 100% juices, which are very high in naturally occurring sugars, may be less desirable than water,^18^ future taxes may want to include such drinks in the tax. We also observed unexpected decreases in nontaxed sparkling waters and coffee drinks. This may have been because some products in these categories were subject to the tax and others were not, leading consumers to assume all of these beverage types were taxed.

Our results also revealed larger declines in taxed beverage volume sales among shoppers in low-income neighborhoods vs those shopping primarily outside low-income neighborhoods. Because individuals with lower incomes tend to consume more sweetened drinks,^19^ they may experience greater health benefits over the long term from these taxes.^20,21^ If those health benefits can translate into greater cost savings for this group over time, as has been shown for tobacco tax policies,^21^ it would help address concerns about the regressive nature of such taxes. In addition, part of the revenue from the Philadelphia beverage tax was used to expand access to high-quality preschool in low-income communities.^8^ One study^22^ found that the revenue spent on these programs exceeds the amount that lower-income groups spend because of the tax. This transfer of funds from higher- to lower-income groups suggests that these taxes can help advance health equity.^22^

Contrary to results from Mexico’s sweetened beverage tax,^23^ we observed similarly large declines in taxed beverage sales among both high- and low-volume taxed beverage purchasers. Consistent with other research,^6^ however, we observed larger reductions for multiserving containers compared with single-serving ones, likely because they had larger absolute increases in price compared with single-serve containers, making the tax more salient.

Our data set was restricted to 1 national pharmacy chain, so it is difficult to know if consumers were reducing their taxed beverage purchases at this pharmacy and simply buying them elsewhere. We did not find evidence that customers were shopping less frequently at the stores in Philadelphia compared with Baltimore as a result of the tax, and the total amount spent per transaction increased. This provides evidence that shoppers were likely not buying drinks elsewhere and also that the tax was not negatively affecting the store’s revenue.

### Strengths and Limitations

This study has several strengths, including its longitudinal design and large sample of objectively measured individual-level purchasing data. Much of the existing work on beverage taxes has reported changes in sales at the store level, which may mask important individual-level patterns. In addition, prior longitudinal sweetened beverage tax evaluations have relied on self-report surveys and receipt collection methods, both of which are prone to measurement and human error.^12,24^

This study also has several limitations. First, our data set was restricted to only 1 national pharmacy chain, though it has many locations in Philadelphia. Second, we were only able to examine purchases, not consumption, though the two are highly correlated.^25^ Third, we were unable to assess other tax effects such as switching to other stores types and cross-border shopping. Fourth, the longitudinal nature of our analyses required us to restrict our data to loyalty cardholders who may differ from non–loyalty cardholders in ways that limit the generalizability of our results. Studies comparing loyalty cardholders to noncardholders in supermarkets have observed some differences in shopping patterns.^26,27,28^ Finally, we lacked sociodemographic information of the cardholders, so we could not examine potential heterogeneity in tax effects based on those characteristics.

## Conclusions

This longitudinal cohort study contributes to the growing body of evidence demonstrating that sweetened beverage taxes significantly reduce purchases of such beverages, which are the top source of added sugar in US diets. These findings suggest these taxes may have public health benefits, including reducing the risk of developing diet-related chronic diseases such as type 2 diabetes, though more research on the health effects of such taxes is needed.

## Notes

Supplement 1. **eTable 1.** Difference-in-Differences Regression Results for Changes in Beverage Price per Ounce Following Implementation of a Beverage Tax in Philadelphia Compared With Providence **eTable 2.** Difference-in-Differences Regression Results for Changes in Beverage Price per Ounce Following Implementation of a Beverage Tax in Philadelphia Compared With Baltimore, Controlling for Store ID, Yearly Quarters, and Store Zip Code–Level Percent Below the Poverty Line **eTable 3.** Difference-in-Differences Regression Results for Individual-Level Changes in Volume of Beverages Purchased Among Purchasers of Beverages Both Before and After Tax, Following Implementation of a Beverage Tax in Philadelphia Compared With Providence **eTable 4.** Difference-in-Differences Regression Results for Individual-Level Changes in Volume of Beverages Purchased Among Purchasers of Beverages Both Before and After Tax, Following Implementation of a Beverage Tax in Philadelphia Compared With Baltimore Controlling for Store ID, Yearly Quarters, and Store Zip Code–Level Percent Below the Poverty Line **eTable 5.** Difference-in-Differences Regression Results for Individual-Level Monthly Changes in Volume of Beverages Purchased Among Purchasers of Beverages Both Before and After Tax Following Implementation of a Beverage Tax in Philadelphia Compared With Baltimore **eTable 6.** Difference-in-Differences Regression Results for Individual-Level Changes in Percentage of Beverages Purchased That Were Taxed vs Nontaxed Before to After Tax Following Implementation of a Beverage Tax in Philadelphia Compared With Baltimore **eTable 7.** Difference-in-Differences Regression Results for Individual-Level Changes in Volume of Beverages Purchased Based on Whether Participants Shopped Primarily in Stores Located in Low-Income Zip Codes vs Non–Low-Income Zip Codes Among Purchasers of Beverages Both Before and After Tax Following Implementation of a Beverage Tax in Philadelphia Compared With Baltimore **eTable 8.** Difference-in-Differences Regression Results for Individual-Level Changes in Volume of Beverages Purchased Based on Whether Consumers Were High- or Low-Volume Purchasers of Taxed Beverages Before Tax Among Purchasers of Beverages Both Before and After Tax Following Implementation of a Beverage Tax in Philadelphia Compared With Baltimore **eTable 9.** Difference-in-Differences Regression Results for Individual-Level Changes in the Percentage of Multiserve vs Single Serve Beverages Purchased by Tax Status Among Purchasers of Beverages Both Before and After Tax Following Implementation of a Beverage Tax in Philadelphia Compared With Baltimore **eFigure.** Parallel Trends Graphs for Philadelphia, Baltimore, and Providence

Supplement 2.
Data Sharing Statement
