# Supplementary material for: Philadelphia Beverage Tax and Association With Prices, Purchasing, and Individual-Level Substitution in a National Pharmacy Chain
Source: JAMA Netw Open. 2023 Jul 13;6(7):e2323200. doi: 10.1001/jamanetworkopen.2023.23200 (PMC10346119; doi:10.1001/jamanetworkopen.2023.23200)
Supplement: Supplement 1. — eTable 1. Difference-in-Differences Regression Results for Changes in Beverage Price per Ounce Following Implementation of a Beverage Tax in Philadelphia Compared With Providence eTable 2. Difference-in-Differences Regression Results for Changes in Beverage Price per Ounce Following Implementation of a Beverage Tax in Philadelphia Compared With Baltimore, Controlling for Store ID, Yearly Quarters, and Store Zip Code–Level Percent Below the Poverty Line eTable 3. Difference-in-Differences Regression Results for Individual-Level Changes in Volume of Beverages Purchased Among Purchasers of Beverages Both Before and After Tax, Following Implementation of a Beverage Tax in Philadelphia Compared With Providence eTable 4. Difference-in-Differences Regression Results for Individual-Level Changes in Volume of Beverages Purchased Among Purchasers of Beverages Both Before and After Tax, Following Implementation of a Beverage Tax in Philadelphia Compared With Baltimore Controlling for Store ID, Yearly Quarters, and Store Zip Code–Level Percent Below the Poverty Line eTable 5. Difference-in-Differences Regression Results for Individual-Level Monthly Changes in Volume of Beverages Purchased Among Purchasers of Beverages Both Before and After Tax Following Implementation of a Beverage Tax in Philadelphia Compared With Baltimore eTable 6. Difference-in-Differences Regression Results for Individual-Level Changes in Percentage of Beverages Purchased That Were Taxed vs Nontaxed Before to After Tax Following Implementation of a Beverage Tax in Philadelphia Compared With Baltimore eTable 7. Difference-in-Differences Regression Results for Individual-Level Changes in Volume of Beverages Purchased Based on Whether Participants Shopped Primarily in Stores Located in Low-Income Zip Codes vs Non–Low-Income Zip Codes Among Purchasers of Beverages Both Before and After Tax Following Implementation of a Beverage Tax in Philadelphia Compared With Baltimore eTable 8. Difference-in-Differences Reg [file jamanetwopen-e2323200-s001.pdf]

Supplementary Online Content

Hua SV, Petimar J, Mitra N, et al. Philadelphia beverage tax and association with prices, purchasing, and individual-level substitution in a national pharmacy chain. *JAMA Netw Open*. 2023;6(7):e2323200. doi:10.1001/jamanetworkopen.2023.23200

eTable 1. Difference-in-Differences Regression Results for Changes in Beverage Price per Ounce Following Implementation of a Beverage Tax in Philadelphia Compared With Providence
eTable 2. Difference-in-Differences Regression Results for Changes in Beverage Price per Ounce Following Implementation of a Beverage Tax in Philadelphia Compared With Baltimore, Controlling for Store ID, Yearly Quarters, and Store Zip Code–Level Percent Below the Poverty Line
eTable 3. Difference-in-Differences Regression Results for Individual-Level Changes in Volume of Beverages Purchased Among Purchasers of Beverages Both Before and After Tax, Following Implementation of a Beverage Tax in Philadelphia Compared With Providence
eTable 4. Difference-in-Differences Regression Results for Individual-Level Changes in Volume of Beverages Purchased Among Purchasers of Beverages Both Before and After Tax, Following Implementation of a Beverage Tax in Philadelphia Compared With Baltimore Controlling for Store ID, Yearly Quarters, and Store Zip Code–Level Percent Below the Poverty Line
eTable 5. Difference-in-Differences Regression Results for Individual-Level Monthly Changes in Volume of Beverages Purchased Among Purchasers of Beverages Both Before and After Tax Following Implementation of a Beverage Tax in Philadelphia Compared With Baltimore
eTable 6. Difference-in-Differences Regression Results for Individual-Level Changes in Percentage of Beverages Purchased That Were Taxed vs Nontaxed Before to After Tax Following Implementation of a Beverage Tax in Philadelphia Compared With Baltimore
eTable 7. Difference-in-Differences Regression Results for Individual-Level Changes in Volume of Beverages Purchased Based on Whether Participants Shopped Primarily in Stores Located in Low-Income Zip Codes vs Non–Low-Income Zip Codes Among Purchasers of Beverages Both Before and After Tax Following Implementation of a Beverage Tax in Philadelphia Compared With Baltimore
eTable 8. Difference-in-Differences Regression Results for Individual-Level Changes in Volume of Beverages Purchased Based on Whether Consumers Were High- or Low-Volume Purchasers of Taxed Beverages Before Tax Among Purchasers of Beverages Both Before and After Tax Following Implementation of a Beverage Tax in Philadelphia Compared With Baltimore

© 2023 Hua SV et al. *JAMA Network Open*.

eTable 9. Difference-in-Differences Regression Results for Individual-Level Changes in the Percentage of Multiserve vs Single Serve Beverages Purchased by Tax Status Among Purchasers of Beverages Both Before and After Tax Following Implementation of a Beverage Tax in Philadelphia Compared With Baltimore
eFigure. Parallel Trends Graphs for Philadelphia, Baltimore, and Providence

This supplementary material has been provided by the authors to give readers additional information about their work.

© 2023 Hua SV et al. *JAMA Network Open*.

eTable 1. Difference-in-Differences Regression Results for Changes in Beverage Price per Ounce Following Implementation of a Beverage Tax in Philadelphia Compared With Providence

	Philadelphia		Providence				
	Pre-tax Mean (SD) cents-per-ounce	Post-tax Mean (SD) cents-per-ounce	Pre-tax Mean (SD) cents-per-ounce	Post-tax Mean (SD) cents-per-ounce	Weighted DID estimate (95% CI)	*p*-value[a]	Percent pass-through[b]
**Taxed Beverages**[c] (n=1,692,611)[d]	8.6 (6.8)	10.3 (7.1)	9.4 (7.2)	9.7 (7.4)	1.6 (1.3, 2.0)	**<.001**	106.7
Soda (n=337,777)	5.8 (4.0)	7.3 (4.0)	6.1 (4.1)	6.2 (3.8)	1.4 (1.1, 1.7)	**<.001**	93.3
Iced Tea & Lemonade (n=308,701)	6.0 (3.5)	6.9 (3.4)	6.6 (4.3)	6.8 (4.7)	0.9 (0.7, 1.1)	**<.001**	60.0
Fruit Drinks (n=231,200)	8.0 (5.5)	8.8 (5.1)	8.1 (5.7)	8.1 (5.4)	0.4 (-0.4, 1.1)	.372	26.7
Sports Drinks (n=230,888)	7.0 (2.3)	9.0 (2.3)	7.1 (2.3)	7.5 (2.2)	1.9 (1.6, 2.2)	**<.001**	126.7
Diet Soda (n=185,704)	5.1 (3.4)	6.6 (3.6)	5.6 (3.6)	5.8 (3.4)	1.4 (1.1, 1.7)	**<.001**	93.3
Sweetened Sparkling Water (n=111,153)	5.9 (5.4)	7.9 (6.5)	6.6 (6.1)	6.3 (5.9)	1.4 (1.1, 1.7)	**<.001**	93.3
Sweetened Coffee (n=50,434)	20.8 (4.2)	23.2 (4.4)	20.8 (4.1)	21.4 (4.1)	1.5 (1.3, 1.6)	**<.001**	100.0
Energy Drinks (n=200,477)	19.5 (5.8)	21.6 (5.3)	19.2 (5.8)	19.9 (5.8)	1.4 (1.0, 1.8)	**<.001**	93.3
**Nontaxed Beverages**[e] (n=748,195)	9.0 (8.1)	9.1 (7.7)	9.4 (8.6)	9.8 (8.10	-0.8 (-2.6, 0.9)	.360	N/A
Regular Water (n=265,580)	4.8 (2.8)	5.3 (3.1)	4.8 (2.8)	5.4 (3.0)	-0.1 (-0.3, 0.02)	.097	N/A
Fruit Juice (n=236,541)	14.6 (9.8)	14.3 (8.7)	15.7 (10.4)	15.3 (9.3)	0.2 (-0.5, 0.8)	.624	N/A
Milk (n=120,213)	6.0 (4.0)	6.4 (4.1)	6.2 (4.5)	7.3 (4.9)	-0.005 (-0.1, 0.1)	.934	N/A
Unsweetened Sparkling Water	6.2 (3.6)	6.9 (6.1)	5.8 (2.4)	6.1 (2.8)	0.03	.886	N/A

© 2023 Hua SV et al. *JAMA Network Open*.

(n=77,972)					(-0.4, 0.5)		
Unsweetened Coffee (n=17,242)	24.0 (8.6)	26.9 (8.3)	23.3 (8.3)	25.4 (8.5)	1.1 (-0.7, 2.9)	.215	N/A

Note. Mean prices are the mean cents-per-ounce per beverage product (SKU), per store, per week. Covariates in all regression models include store ID. Providence is comparison city.

[a]Bolded values indicate statistical significance after applying the Holm-Bonferroni correction for multiple testing on families of outcome (total taxed beverages [1 test], taxed beverage subcategories [8 tests], total nontaxed beverages [1 test], nontaxed beverage subcategories [5 tests])

[b]The percent pass-through was calculated for taxed beverages as the difference-in-differences point estimate divided by 1.5 cents/oz.

[c]Overall taxed category does not sum up to the composite categories because it also includes milk alternatives and other taxed beverages.

[d]Ns represent the number of unique week by store ID by SKU combinations over 104 weeks pre-tax and 52 weeks post-tax. In total, there were 1,164 unique SKUs in this dataset across 58 unique stores.

[e]Overall nontaxed category does not sum up to the composite categories because it also includes milk alternatives, flavored waters, iced tea or lemonade, sports drinks, and other nontaxed beverages.

© 2023 Hua SV et al. *JAMA Network Open*.

eTable 2. Difference-in-Differences Regression Results for Changes in Beverage Price per Ounce Following Implementation of a Beverage Tax in Philadelphia Compared With Baltimore, Controlling for Store ID, Yearly Quarters, and Store Zip Code–Level Percent Below the Poverty Line

	Philadelphia		Baltimore				
	Pre-tax Mean (SD) cents-per-ounce	Post-tax Mean (SD) cents-per-ounce	Pre-tax Mean (SD) cents-per-ounce	Post-tax Mean (SD) cents-per-ounce	Weighted DID estimate (95% CI)	*p*-value[a]	Percent pass-through[b]
**Taxed Beverages**[c] (n=2,058,561)[d]	8.6 (6.8)	10.3 (7.1)	8.2 (6.5)	8.6 (6.9)	1.6 (1.3, 2.0)	**<.001**	106.7
Soda (n=439,834)	5.8 (4.0)	7.3 (4.0)	5.6 (4.0)	5.8 (3.9)	1.4 (1.1, 1.7)	**<.001**	93.3
Iced Tea & Lemonade (n=404,144)	6.0 (3.5)	6.9 (3.4)	6.0 (3.3)	6.3 (3.4)	0.9 (0.7, 1.1)	**<.001**	60.0
Fruit Drinks (n=284,624)	8.0 (5.4)	8.8 (5.1)	7.6 (5.0)	7.3 (4.6)	0.8 (0.6, 1.0)	**<.001**	53.3
Sports Drinks (n=262,835)	7.0 (2.3)	9.0 (2.3)	7.1 (2.1)	7.6 (2.1)	1.8 (1.5, 2.2)	**<.001**	120.0
Diet Soda (n=219,423)	5.1 (3.4)	6.6 (3.6)	5.3 (3.5)	5.7 (3.4)	1.5 (1.1, 1.9)	**<.001**	100.0
Sweetened Sparkling Water (n=123,298)	5.9 (5.4)	7.9 (6.5)	5.6 (4.7)	5.4 (4.9)	1.5 (1.2, 1.8)	**<.001**	100.0
Sweetened Coffee (n=61,368)	20.8 (4.2)	23.2 (4.4)	20.4 (4.0)	21.3 (4.1)	1.1 (0.8, 1.4)	**<.001**	73.3
Energy Drinks (n=227,767)	19.5 (5.8)	21.6 (5.3)	19.5 (6.0)	21.2 (6.6)	0.2 (-0.4, 0.8)	.514	N/A
**Nontaxed Beverages**[e] (n=852,743)	9.0 (8.1)	9.1 (7.7)	9.1 (8.3)	9.0 (7.8)	0.1 (-0.7, 0.9)	.643	N/A
Regular Water (n=313,117)	4.8 (2.8)	5.3 (3.1)	4.8 (2.8)	5.2 (3.1)	-0.02 (-0.2, 0.1)	.752	N/A
Fruit Juice (n=262,724)	14.5 (9.8)	14.3 (8.7)	14.9 (10.6)	14.7 (9.5)	0.2 (-0.3, 0.7)	.465	N/A
Milk (n=141,416)	6.1 (4.1)	6.4 (4.1)	6.5 (4.3)	6.8 (4.8)	0.04 (-0.1, 0.1)	.434	N/A

© 2023 Hua SV et al. *JAMA Network Open*.

Unsweetened Sparkling Water (n=78,666)	6.2 (3.6)	6.9 (6.1)	6.4 (2.9)	6.1 (2.9)	0.6 (0.2, 1.0)	**<.01**	N/A
Unsweetened Coffee (n=17,410)	24.0 (8.6)	26.9 (8.3)	24.5 (9.1)	28.3 (10.4)	-1.9 (-3.7, -0.1)	<.05	N/A

Note. Mean prices are the mean cents-per-ounce per beverage product (SKU), per store, per week. Covariates in all regression models include store ID, yearly quarters, and store zip code-level percent below the poverty line. Baltimore is comparison city.

[a]Bolded values indicate statistical significance after applying the Holm-Bonferroni correction for multiple testing on families of outcome (total taxed beverages [1 test], taxed beverage subcategories [8 tests], total nontaxed beverages [1 test], nontaxed beverage subcategories [5 tests])

[b]The percent pass-through was calculated for taxed beverages as the difference-in-differences point estimate divided by 1.5 cents/oz.

[c]Overall taxed category does not sum up to the composite categories because it also includes milk alternatives and other taxed beverages.

[d]Ns represent the number of unique week by store ID by SKU combinations over 104 weeks pre-tax and 52 weeks post-tax. In total, there were 1,188 unique SKUs in this dataset across 75 unique stores.

[e]Overall nontaxed category does not sum up to the composite categories because it also includes milk alternatives, flavored waters, iced tea or lemonade, sports drinks, and other nontaxed beverages.

© 2023 Hua SV et al. *JAMA Network Open*.

eTable 3. Difference-in-Differences Regression Results for Individual-Level Changes in Volume of Beverages Purchased Among Purchasers of Beverages Both Before and After Tax, Following Implementation of a Beverage Tax in Philadelphia Compared With Providence[a]

	Philadelphia		Providence			
	Pre-tax Mean (SD) ounces purchased	Post-tax Mean (SD) ounces purchased	Pre-tax Mean (SD) ounces purchased	Post-tax Mean (SD) ounces purchased	Relative % Change (95% CI)	*p*-value[b]
**Taxed Beverages**[c] (n=3,196,535)[d]	73.0 (118.0)	59.1 (103.0)	63.0 (118.4)	60.4 (103.7)	-8.4 (-8.8, -8.0)	**<.001**
Soda (n=794,474)	95.5 (145.7)	75.3 (137.3)	95.3 (197.2)	86.0 (170.6)	-7.6 (-8.5, -6.6)	**<.001**
Iced Tea & Lemonade (n=832,865)	54.6 (79.5)	47.2 (62.6)	42.4 (44.5)	44.2 (42.5)	-11.8 (-12.6, -10.9)	**<.001**
Fruit Drinks (n=470,717)	49.2 (71.4)	42.5 (58.1)	43.8 (46.9)	45.3 (44.8)	-10.3 (-11.3, -9.4)	**<.001**
Sports Drinks (n=436,738)	43.1 (35.4)	39.4 (26.7)	40.6 (30.4)	42.0 (31.3)	-7.3 (-8.1, -6.4)	**<.001**
Diet Soda (n=431,595)	96.32 (122.9)	76.0 (94.7)	80.9 (109.1)	74.6 (99.9)	-8.3 (-9.4, -7.2)	**<.001**
Sweetened Sparkling Water (n=277,255)	54.2 (65.1)	51.6 (64.5)	40.2 (44.8)	42.5 (48.3)	-4.0 (-5.4, 2.6)	**<.001**
Sweetened Coffee (n=90,614)	18.5 (12.4)	17.0 (10.0)	17.6 (10.2)	17.8 (10.9)	-7.5 (-9.0, -5.9)	**<.001**
Energy Drinks (n=329,276)	21.4 (14.9)	20.8 (14.4)	20.9 (13.7)	20.9 (13.8)	-2.5 (-3.3, -1.6)	**<.001**
**Nontaxed Beverages**[e] (n= 2,388,308)	142.3 (249.9)	134.1 (243.1)	133.4 (232.1)	123.6 (222.9)	1.3 (0.7, 1.9)	**<.001**
Regular Water (n=1,075,972)	227.1 (341.9)	206.4 (334.1)	212.7 (330.2)	194.6 (320.5)	-0.02 (-0.9, 0.9)	.970
Fruit Juice (n=407,713)	37.4 (34.0)	39.1 (32.6)	37.0 (33.7)	36.1 (28.6)	5.7 (4.4, 6.9)	**<.001**
Milk (n=765,588)	87.0 (57.7)	85.4 (56.8)	93.6 (64.5)	89.4 (63.5)	-0.5 (-0.5, 0.6)	.871

© 2023 Hua SV et al. *JAMA Network Open*.

Unsweetened Sparkling Water (n=218,413)	44.5 (39.9)	50.3 (42.0)	40.5 (36.6)	44.7 (40.0)	-0.1 (-1.6, 1.5)	.919
Unsweetened Coffee (n=44,405)	12.9 (8.5)	12.7 (8.4)	12.6 (7.1)	13.1 (6.7)	-6.9 (-9.4, -4.3)	**<.001**

Note. Mean ounces purchased are raw means per card, per transaction. Regression models only include cardholder IDs that had beverage transactions in both the pre- and post- tax periods. Providence is comparison city.

[a]Cross-classified mixed models were used to produce results for absolute changes in volume. Generalized linear mixed models with a log link were used to produce results for relative percent changes in volume.

[b]Bolded values indicate statistical significance after applying the Holm-Bonferroni correction for multiple testing on families of outcome (total taxed beverages [1 test], taxed beverage subcategories [8 tests], total nontaxed beverages [1 test], nontaxed beverage subcategories [5 tests])

[c]The number of observations in the overall taxed category are fewer than the sum of the composite categories because it sums the volume of all taxed beverages purchased in an individual transaction, creating a single observation if there were multiple taxed beverage types included in the overall analysis.

[d]Ns represent the number of unique transactions over 104 weeks pre-tax and 52 weeks post-tax.

[e]The number of observations in the overall nontaxed category are fewer than the sum of the composite categories because it sums the volume of all nontaxed beverages purchased in an individual transaction, creating a single observation if there were multiple nontaxed beverage types included in the overall analysis.

© 2023 Hua SV et al. *JAMA Network Open*.

eTable 4. Difference-in-Differences Regression Results for Individual-Level Changes in Volume of Beverages Purchased Among Purchasers of Beverages Both Before and After Tax, Following Implementation of a Beverage Tax in Philadelphia Compared With Baltimore Controlling for Store ID, Yearly Quarters, and Store Zip Code–Level Percent Below the Poverty Line

	Philadelphia		Baltimore			
	Pre-tax Mean (SD) ounces purchased	Post-tax Mean (SD) ounces purchased	Pre-tax Mean (SD) ounces purchased	Post-tax Mean (SD) ounces purchased	Adjusted[a] DID estimate (95% CI)	*p*-value[b]
**Taxed Beverages[c]** (n= 3582799)[d]	73.0 (118.0)	59.1 (102.9)	98.8 (148.1)	81.5 (144.6)	-6.5 (-7.0, -5.9)	**<.001**
Soda (n=981,503)	95.5 (145.7)	75.3 (137.2)	120.4 (175.7)	103.4 (149.6)	-7.0 (-8.1, -5.8)	**<.001**
Iced Tea & Lemonade (n=968,646)	54.5 (79.5)	47.2 (62.6)	53.3 (78.8)	56.2 (99.6)	-8.3 (-9.0, -7.7)	**<.001**
Fruit Drinks (n=541,626)	49.2 (71.4)	42.4 (57.9)	50.5 (76.4)	55.3 (123.0)	-8.5 (-9.3, -7.6)	**<.001**
Sports Drinks (n=442,942)	43.1 (35.4)	39.4 (26.8)	43.8 (34.6)	42.5 (31.1)	-2.6 (-3.1, -2.1)	**<.001**
Diet Soda (n=481,112)	96.3 (122.8)	75.9 (94.7)	122.5 (157.4)	102.1 (134.0)	-2.6 (-4.0, -1.3)	**<.001**
Sweetened Sparkling Water (n=280,944)	54.2 (65.1)	51.6 (64.5)	45.6 (54.7)	46.5 (55.3)	-2.7 (-3.8, -1.6)	**<.001**
Sweetened Coffee (n=98,528)	18.5 (12.4)	17.0 (10.0)	19.7 (15.9)	19.7 (13.9)	-1.2 (-1.5, -0.8)	**<.001**
Energy Drinks (n=339,537)	21.4 (14.9)	20.8 (14.3)	21.0 (14.4)	21.0 (12.7)	-0.5 (-0.7, -0.2)	**<.001**
**Nontaxed Beverages[e]** (n= 2415620)	142.3 (250.0)	134.0 (243.0)	129.4 (228.1)	118.7 (222.8)	1.8 (0.2, 3.3)	**<.05**
Regular Water (n=1,099,099)	227.0 (342.1)	206.3 (334.0)	203.4 (316.5)	177.6 (307.4)	5.4 (2.5, 8.4)	**<.001**
Fruit Juice (n=420,505)	37.4 (34.0)	39.1 (32.6)	38.6 (51.6)	39.3 (49.1)	0.4 (-0.2, 0.9)	.22
Milk (n=761,829)	86.9 (57.7)	85.4 (56.8)	90.8 (88.7)	83.2 (74.9)	2.7 (2.1, 3.3)	**<.001**

© 2023 Hua SV et al. *JAMA Network Open*.

Unsweetened Sparkling Water (n=208,990)	44.5 (39.9)	50.3 (42.0)	39.2 (36.9)	49.2 (44.3)	-1.7 (-2.8, -0.6)	**<.01**
Unsweetened Coffee (n=46,287)	12.9 (8.4)	12.7 (8.4)	12.7 (7.1)	12.8 (7.6)	-0.4 (-0.8, 0.09)	.11

Note. Mean ounces purchased are raw means per card, per transaction. Regression models only include cardholder IDs that had beverage transactions in both the pre- and post- tax periods. Baltimore is comparison city.

[a]Bolded values indicate statistical significance after applying the Holm-Bonferroni correction for multiple testing on families of outcome (total taxed beverages [1 test], taxed beverage subcategories [8 tests], total nontaxed beverages [1 test], nontaxed beverage subcategories [5 tests]

[b]Covariates included yearly quarters and store zip code-level percent below the poverty line.

[c]The number of observations in the overall taxed category are fewer than the sum of the composite categories because it sums the volume of all taxed beverages purchased in an individual transaction, creating a single observation if there were multiple taxed beverage types included in the overall analysis.

[d]Ns represent the number of unique transactions over 104 weeks pre-tax and 52 weeks post-tax.

[e]The number of observations in the overall nontaxed category are fewer than the sum of the composite categories because it sums the volume of all nontaxed beverages purchased in an individual transaction, creating a single observation if there were multiple nontaxed beverage types included in the overall analysis.

© 2023 Hua SV et al. *JAMA Network Open*.

eTable 5. Difference-in-Differences Regression Results for Individual-Level Monthly Changes in Volume of Beverages Purchased Among Purchasers of Beverages Both Before and After Tax Following Implementation of a Beverage Tax in Philadelphia Compared With Baltimore[a]

	Relative % Change (95% CI)	*p*-value
**Taxed Beverages** (n=2,117,355)	-8.2 (-8.7, -7.8)	**<.001**
**Nontaxed Beverages** (n=)	1.0 (0.3, 1.7)	**<.01**

[a]Generalized linear mixed models with a log link were used for relative percent changes in volume.
Baltimore is comparison city.

© 2023 Hua SV et al. *JAMA Network Open*.

eTable 6. Difference-in-Differences Regression Results for Individual-Level Changes in Percentage of Beverages Purchased That Were Taxed vs Nontaxed Before to After Tax Following Implementation of a Beverage Tax in Philadelphia Compared With Baltimore[a]

	DID estimate (95% CI)	Relative % Change (95% CI)	*p*-value
**Taxed Beverages** (n=627,164)	-0.1 (-0.2, -0.1)	-13.4 (-14.2, -12.6)	**<.001**
**Nontaxed Beverages** (n= 627,164)	0.09 (0.08, 0.1)	9.3 (7.7, 10.7)	**<.001**

[a]Data were aggregated up to the pre and post-tax level
Baltimore is comparison city.

© 2023 Hua SV et al. *JAMA Network Open*.

eTable 7. Difference-in-Differences Regression Results for Individual-Level Changes in Volume of Beverages Purchased Based on Whether Participants Shopped Primarily in Stores Located in Low-Income Zip Codes vs Non–Low-Income Zip Codes Among Purchasers of Beverages Both Before and After Tax Following Implementation of a Beverage Tax in Philadelphia Compared With Baltimore[a,b]

	DID estimate (95% CI)	Relative % Change (95% CI)	Triple DID estimate (95% CI)	Triple DID *p*-value[c]
**Taxed Beverages** (n=3,582,799)[d]				
Low income (n=153,190)	-0.1 (-0.2, -0.1)	-12.7 (-14.3, -10.9)	-0.1 (-0.1, -0.1)	**<.001**
Other income (n=3,429,609)	-0.1 (-0.1, -0.1)	-7.3 (-7.6, -7.0)		
**Nontaxed Beverages** (n=2,415,620)				
Low income (n=103,911)	0.03 (-0.001, 0.1)	3.2 (-0.1, 6.6)	0.01 (-0.02, 0.04)	.495
Other income (n=2,311,709)	0.02 (0.01, 0.02)	1.5 (0.9, 2.2)		

[a]Low-income zip codes are those with ≥30% of the population at or below federal poverty level based on the 2019 American Community Survey
[b]Prices of beverages did not differ based on a store's location in low- versus other-income zip code
[c]The triple DID *p*-value indicates whether there is a statistically significant difference between the estimates for low- and other-income within the taxed and nontaxed categories
[d]Ns represent the number of transactions made by shoppers who purchased primarily from stores located in low/other-income stores over 104 weeks pre-tax and 52 weeks post-tax
Baltimore is comparison city.

© 2023 Hua SV et al. *JAMA Network Open*.

eTable 8. Difference-in-Differences Regression Results for Individual-Level Changes in Volume of Beverages Purchased Based on Whether Consumers Were High- or Low-Volume Purchasers of Taxed Beverages Before Tax Among Purchasers of Beverages Both Before and After Tax Following Implementation of a Beverage Tax in Philadelphia Compared With Baltimore

	DID estimate (95% CI)	Relative % Change (95% CI)	Triple DID estimate (95% CI)	Triple DID *p*-value[a]
**Taxed Beverages** (n=3,634,736)				
High volume taxed beverage purchasers[b] (n=2,409,900)[c]	-0.1 (-0.1, -0.1)	-7.6 (-8.0, -7.2)	-0.002 (-0.01, 0.01)	.592
All other taxed beverage purchasers (n=1,224,836)	-0.1 (-0.1, -0.1)	-8.9 (-9.4, -8.3)		
**Nontaxed Beverages** (n= 2,443,752)				
High volume taxed beverage purchasers (n=1,297,512)	0.01 (0.001, 0.02)	0.9 (0.1, 1.8)	-0.003 (-0.01, 0.01)	.596
All other taxed beverage purchasers (n=1,146,240)	0.01 (0.004, 0.02)	1.2 (0.4, 2.0)		

[a]The triple DID *p*-value indicates whether there is a statistically significant difference between the estimates for low-volume taxed beverage purchasers and high-volume taxed beverage purchasers within the taxed and nontaxed categories

[b]High- and low- volume taxed beverage purchasers were those who purchased $\geq$75th percentile and <75th percentile of taxed beverage ounces in the pretax period

[c]Ns represent the number of transactions that low/high volume taxed beverage purchasers made over 104 weeks pre-tax and 52 weeks post-tax

Baltimore is comparison city.

© 2023 Hua SV et al. *JAMA Network Open*.

eTable 9. . Difference-in-Differences Regression Results for Individual-Level Changes in the Percentage of Multiserve vs Single Serve Beverages Purchased by Tax Status Among Purchasers of Beverages Both Before and After Tax Following Implementation of a Beverage Tax in Philadelphia Compared With Baltimore[a]

	DID estimate (95% CI)	Relative % Change (95% CI)	Triple DID estimate (95% CI)	Triple DID *p*-value[b]
**Taxed Beverages**[c] (n=968,119)				
Single serve (n=444,311)	-0.03 (-0.4, -0.02)	-3.2 (-4.3, -2.1)	-0.2 (-0.2, -0.2)	**<.001**
Multi-serve (n=523,808)	-0.2 (-0.2, -0.2)	-18.4 (-19.3, -17.4)		
**Nontaxed Beverages**[d] (n=968,119)				
Single serve (n=444,311)	-0.02 (-0.04, 0.002)	-1.8 (-3.7, 0.2)	0.2 (0.1, 0.2)	**<.001**
Multi-serve (n=523,808)	0.1 (0.1, 0.2)	15.4 (13.7, 17.0)		

[a]Data were aggregated up to the pre- and post-tax level
[b]The triple DID *p*-value indicates whether there is a statistically significant difference between the estimates for single-serve and multi-serve beverages within the taxed and nontaxed categories
[c]Beverages classified as “other” (i.e., RACC could not be readily determined) were dropped (1.06%)
[d]Beverages classified as “other” (i.e., RACC could not be readily determined) were dropped (0.24%)
Baltimore is comparison city.

© 2023 Hua SV et al. *JAMA Network Open*.

eFigure. Parallel Trends Graphs for Philadelphia, Baltimore, and Providence

(a) weekly average price-per-ounce of taxed beverages by city; (b) weekly average price-per-ounce of nontaxed beverages by city; (c) weekly average volume sold of taxed beverages by city; and (d) weekly average volume sold of nontaxed beverages by city

(a)

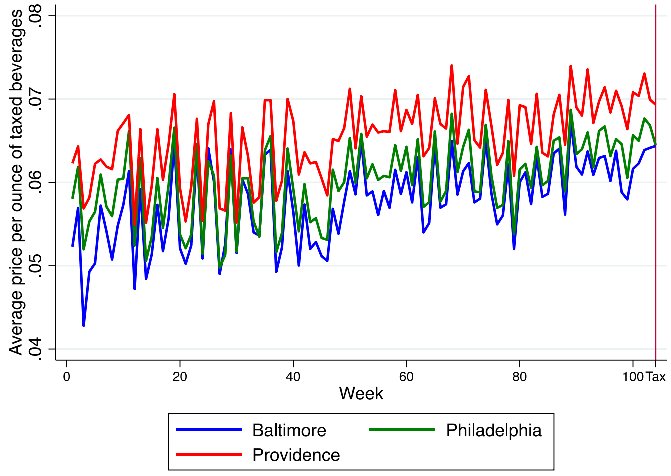

(b)

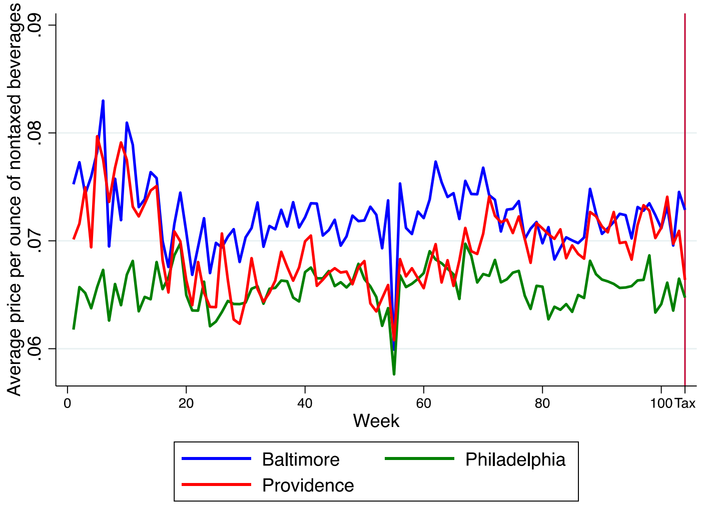

© 2023 Hua SV et al. *JAMA Network Open*.

(c)

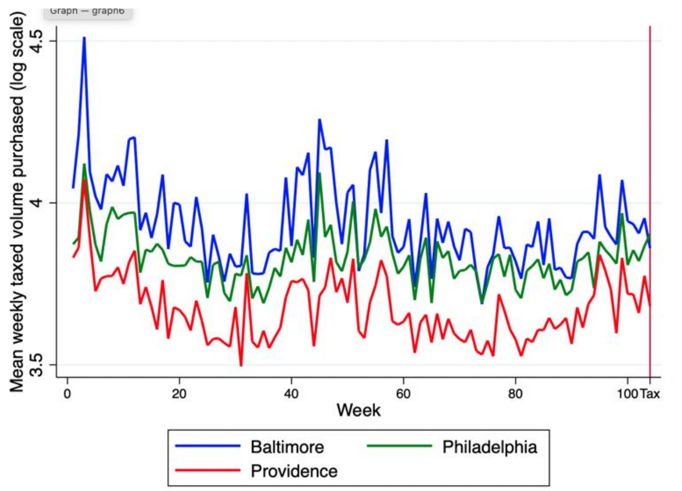

(d)

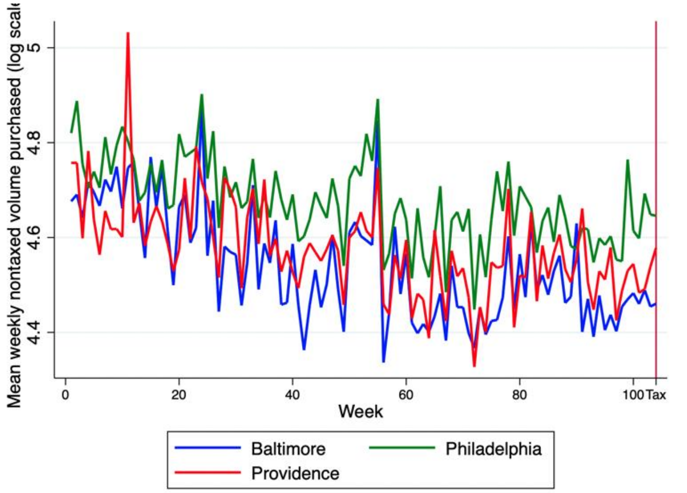

© 2023 Hua SV et al. *JAMA Network Open*.
