# Supplementary material for: Philadelphia Beverage Tax and Association With Prices, Purchasing, and Individual-Level Substitution in a National Pharmacy Chain
Source: JAMA Netw Open. 2023 Jul 13;6(7):e2323200. doi: 10.1001/jamanetworkopen.2023.23200 (PMC10346119; doi:10.1001/jamanetworkopen.2023.23200)
Supplement: Supplement 2. — Data Sharing Statement [file jamanetwopen-e2323200-s002.pdf]

Data Sharing Statement

Hua. Philadelphia Beverage Tax and Association With Prices, Purchasing, and Individual-Level Substitution in a National Pharmacy Chain. *JAMA Netw Open*. Published July 13, 2023. doi:10.1001/jamanetworkopen.2023.23200

Data
Data available: No

Additional Information
Explanation for why data not available: Currently, we do not have ethics, institutional, or retailer (the entity that shared the data with us) permissions in place for data sharing outside of the study team and close collaborators.
